# Analysis of Dry Needling Combined with an Exercise Program in the Treatment of Knee Osteoarthritis: A Randomized Clinical Trial

**DOI:** 10.3390/jcm13237157

**Published:** 2024-11-26

**Authors:** Aida Agost-González, Isabel Escobio-Prieto, Cristo Jesús Barrios-Quinta, María de los Ángeles Cardero-Durán, Luis Espejo-Antúnez, Manuel Albornoz-Cabello

**Affiliations:** 1 Department of Physiotherapy, Faculty of Nursing, Physiotherapy and Podiatry, University of Seville, 41009 Seville, Spain; aidaagost97@gmail.com (A.A.-G.); malbornoz@us.es (M.A.-C.); 2 Department of Physiotherapy, Faculty of Nursing, Physiotherapy and Podiatry, IBiS, Institute of Biomedicine of Seville, Neurological Physiotherpy, Innovative Neurorehabilitation and Neurodevelopment Disorders, CTS-1137, University of Seville, 41009 Seville, Spain; 3 Physiotherapy Unit, Andalusian Health Service, 41005 Seville, Spain; cristo.barrios@gmail.com; 4 Department of Medical-Surgical Therapy, Medicine Faculty, University of Extremadura, 06071 Badajoz, Spain; mcarderod@unex.es (M.d.l.Á.C.-D.); luisea@unex.es (L.E.-A.)

**Keywords:** knee osteoarthritis, dry needling, popliteus muscle, therapeutic exercise, pain, functionality

## Abstract

**Background:** Therapeutic exercise is recommended for people with knee osteoarthritis (OA), although it could be complemented with other treatments such as dry needling (DN). The purpose of this study was to evaluate and compare the resulting data on pain, functionality, strength and range of motion in subjects with knee osteoarthritis after being treated with a specific therapeutic physical exercise program alone or in combination with the DN technique in the popliteus muscle. **Methods**: A total of 33 participants were randomly assigned to two groups: the dry needling plus therapeutic physical exercise group (*n* = 15) and the therapeutic physical exercise alone group (*n* = 18). Both groups received the same exercise protocol, and the dry-needling group conducted three sessions of this technique over 3 weeks. **Results**: Variables such as pain, functionality, neuropathic pain, stiffness, strength, range of motion, pain catastrophizing and kinesiophobia were evaluated before and after the intervention, as well as at a follow-up 3 months after the intervention. Significant differences were observed between the two groups in pain intensity, stiffness, functionality, pain catastrophizing and kinesiophobia (*p* < 0.001). **Conclusions**: The combination of dry needling targeting the popliteus muscle and therapeutic physical exercise showed better results in terms of pain, functionality and strength compared to therapeutic physical exercise alone, especially after the intervention.

## 1. Introduction

Osteoarthritis (OA) is the most prevalent degenerative joint disease in the general population [1], and it often affects the elderly more [2]. Knee OA is the most common type, affecting 265 million people worldwide [3]. About 40% of adults over 45 years of age exhibit radiographic evidence of knee OA [4], making it a major cause of disability [5], which leads to a decrease in the quality of life for those affected [3].

Knee OA causes injuries to bones, joints, ligaments, synovial membrane, joint capsules and periarticular muscles [6].

Treatment for knee OA can be conservative or surgical. Conservative therapies are non-invasive treatments and include education, exercise, weight reduction for people who are overweight or obese and the use of orthotics [7]. Among the non-surgical treatments, which do not use surgery for treatment, medication is usually the first choice for relieving the symptoms of osteoarthritis [8].

Clinical guidelines recommend interventions other than surgery, including exercise, as the first line of treatment for knee OA [9]. Exercise is highly recommended for people with knee OA due to its benefits in relation to pain, function and quality of life [10].

A relationship has been observed between the presence of myofascial trigger points in periarticular muscles of the knee and muscle pain associated with knee OA [11]. 

A trigger point is an area of hypersensitivity located in a tense band of a muscle, the activation of which can trigger motor and sensory disturbances [12].

Dry needling (DN) is an intervention that uses a fine filiform needle to stimulate myofascial trigger points for the treatment of musculoskeletal disorders. The primary goal of DN is to improve the function of the muscle [13].

It has been observed that performing the DN technique combined with stretching exercises in subjects with knee OA leads to significant improvements in terms of pain, functionality and range of motion [14]. Moreover, the effects of DN are longer lasting compared to the intake of oral drugs [15]. In addition, three sessions of DN targeting the muscles around the hip or knee can improve function, sensation, balance and pain in patients with knee OA in the short term [16].

The involvement of the popliteus muscle in knee pain is often underestimated, even if the distension of this muscle is the cause of some pain in this joint [17,18]. It is also considered that the presence of trigger points in this muscle may represent a cause of diffuse knee pain [12].

The popliteus muscle is a posterolateral structure that helps to control external rotation, varus and posterior displacement of the tibia [19]. Active trigger points in the popliteal muscle have been identified in 17% of people with symptomatic knee OA. These trigger points may produce pain, muscle weakness and decreased range of motion [20]; they can also induce stiffness [21].

The popliteus muscle is a part of the deep musculature of the popliteal fossa, so it cannot be accessed through manual therapy due to the superficial muscular structures and vasculonervous bundle. Therefore, the most accessible treatment method is dry needling [22].

Although the use of DN in deep musculature has been controversial due to the possibility of puncturing vasculonervous structures [23], a cadaver study in 2021 showed a safe and effective way to perform the DN technique on the popliteus muscle [24].

Due to the efficacy of DN [15,16] and therapeutic exercise [9,10] in subjects with knee OA, this study aims to combine both therapies, as suggested by previous studies [14,25].

Taking into account the effectiveness of exercise as a treatment for knee OA and the usefulness of DN in alleviating its symptoms, the aim of this study was to evaluate and compare the resulting effects on pain, functionality, strength and range of motion in subjects with knee OA after treatment with a specific exercise program alone or in combination with DN technique in the popliteus muscle.

## 2. Materials and Methods

### 2.1. Study Design

The present study is a randomized, prospective, single-blinded clinical trial, with the participants and the investigator in charge of collecting the data remaining blind to the treatment applied to each subject. This study was approved by the Ethics Committee of the Virgen Macarena and Virgen del Rocio University Hospitals and was registered in the ClinicalTrials.gov Clinical Trials Registry (code: 0808-N-22). The research protocol was designed following the Declaration of Helsinki and the standards established in the CONSORT (Consolidated Standards of Reporting Trial) statement. In this way, the participants were informed about this study and their rights prior to signing the informed consent form.

### 2.2. Sample Size

Sample size calculation was based on detecting the following: (1) a 15% improvement in self-perceived pain intensity [26]; (2) a difference of >9 points in the LEFS score at inter-group comparison after the treatment [27]; and >10 points in the Kujala score. Taking into account a one-tail hypothesis, for repeated measures ANOVA, within–between interactions, an alpha value of 0.05, a desired power of 80% and a medium effect size (r^2^ = 0.24), 30 participants were needed (G*Power, version 3.1.9.2). To enhance the statistical power, the target sample size was adjusted to nearly 36 participants. A similar sample size has been observed in studies of patients with knee OA [16,28] and in studies on the DN technique [14,29].

### 2.3. Participants

In this study, 36 subjects were initially recruited, of whom 33 subjects were over 18 years of age, had a diagnosis of knee OA, unilateral or bilateral, and attended physiotherapy at the “El Cachorro” Health Center in Seville. Those who did not give their consent to participate, minors, and those subjects with Baker´s cyst in the knee, knee prostheses, surgical intervention in the area, or treatment in less than 6 months in the study area were excluded. Although a radiological analysis of each participant was not conducted, most participants were referred with grade 1 osteoarthritis, followed by grades 2, 4 and 3.

### 2.4. Randomization and Blinding

The researcher responsible for collecting data from the participants was blinded. The random allocation was conducted using EPIDAT software version 3.1, considering a 1:1 ratio to assign participants to the control group (supervised exercise] and the experimental group (supervised exercise and DN). A different researcher distributed the allocation to participants using opaque envelopes, i.e., being blinded to their content. To prevent participants from knowing the treatment given to the other group, the program instructions were given to each group separately.

### 2.5. Outcomes

Clinical and demographic information was obtained from all participants. The variables to be studied were the same for both groups: the WOMAC questionnaire (Western Ontario and McMaster Universities Osteoarthritis Index questionnaire) in its reduced Spanish version was used to measure symptomatology and disability [30].

The maximum pain experienced by patients in the last 24 h was measured with the visual analog scale (VAS), which consists of a horizontal line from 0 to 100 mm, where a score of 0 mm would mean “no pain” and 100 mm would mean “maximum intolerable pain” [31]. The DN4 questionnaire was used as the evaluation scale for neuropathic pain, consisting of 10 items with a score of 1 per item; a total score equal to or higher than 4 indicates neuropathic pain [32]. Functional disability was assessed with the Kujula Score Test, which consists of 13 questions related to pain, physical alterations, possible limitations in functional capacity and the ability to participate in sports. Each question has several possible answers; the maximum score is 100 points, and the minimum is 0 points [33]. The Lower Extremity Functional Scale (LEFS) was used, which consists of 20 items, each with a maximum score of 4. The maximum possible score is 80 points, which indicates a high functional level [27]. Data from the IPAQ (International Physical Activity Questionnaire) were also recorded [34].

Finally, knee flexion and extension joint balance were passively determined with a conventional two-legged goniometer, which has been shown to have high intra- and inter-test reliability for flexion and extension [35], and the extension force was assessed by a manual sphygmomanometer, measured in millimeters of mercury (mmhg) [36]. To evaluate fear in relation to pain, the TSK-11SV (Tampa Scale of Kinesiophobia-11) questionnaire was administered [37], and pain catastrophizing was measured using the Pain Catastrophizing Scale questionnaire [38]. In addition, the 6MWT (6-Minute Walk Test) [39] and Timed-up and Go (TUG) tests were carried out [40].

### 2.6. Interventions

The study was carried out in several phases: in the initial phase, a meeting was held at the center with the patients selected for the study, during which they were provided with detailed information about the research, and any doubts raised were resolved. Likewise, it was verified that each patient adequately met the established inclusion and exclusion criteria. Subsequently, they were individually given the informed consent form specifically prepared for the present study. Next, we proceeded with the preparation of the clinical history of physical therapy, recording only the information relevant to the research objectives. After the initial phase, the 36 participants were randomly assigned to one of two groups: the experimental group (EG), which received the DN technique on the popliteus muscle combined with therapeutic exercise, or the control group (CG), which performed only therapeutic exercise without puncture. The allocation was carried out in a 1:1 ratio, in a concealed manner, using a website (http://www.randomization.com (accessed on 29 September 2024)), before the start of data collection. The participants were also given opaque envelopes with the group assignment, without them being able to recognize the result. In turn, a blinded researcher collected the study measurements.

The EG had 3 sessions of DN intervention distributed over three weeks [16,29], combined with the performance of a specific exercise protocol for this condition, which was performed 4 times per week.

The choice of 3 DN sessions was based on Mohammadreza Farazdaghi’s study on dry needling in subjects with knee OA [16] and Christian Haser’s study on dry needling of knee extensor muscles [29].

The CG was summoned the same number of times as the experimental group using a simulated DN intervention, in addition to the same exercise protocol.

The interventions are explained below.

**Figure 1:**
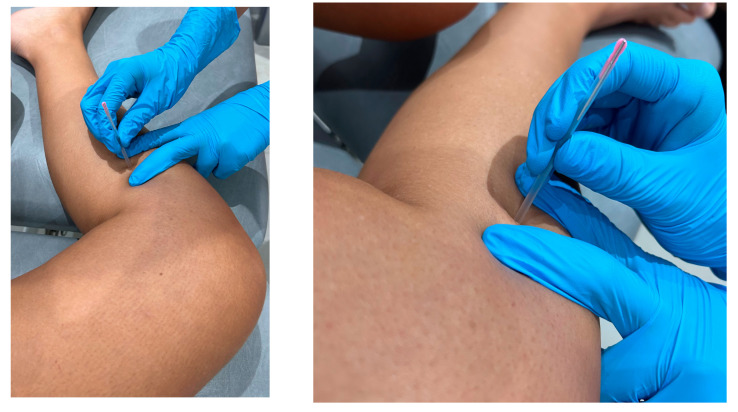
Image of the simulated puncture procedure and the procedure prior to the DN technique performed.

For the DN technique, the patient was placed in lateral decubitus on the affected knee and flexed at approximately 90°; the skin was then cleaned with a suitable antiseptic solution, and the needle was inserted from the medial to lateral direction in the upper third of the tibia, keeping as close as possible to the posterior aspect of the tibia bone up to a depth that was considered the popliteus muscle, thus isolating the vasculonervous bundle [24] (Figure 1). The needle was removed after puncture and deposited in a needle container.

Sterile, single-use needles of 0.30 mm in diameter and 50 mm in length were used. Prior to the insertion of the needle, pressure was applied with the insertion tube to minimize the pain of the puncture [41].

In the simulated DN intervention, after cleaning the skin, as in the EG, pressure was applied with the insertion tube, causing a prick sensation without penetrating the skin. 

The exercise protocol (available in the Supporting information section of the ACR journal website at https://acrjournals.onlinelibrary.wiley.com/doi/ftr/10.1002/acr.22744 (accessed on 3 October 2024)) was standardized across the population. It consisted of 8 strengthening exercises for the quadriceps, hamstrings and hip abductor muscles, which were performed 4 times per week for 3 months [42]. These exercises consisted of knee extension and flexion, hip abduction, isometric exercises for the knee extensor muscles and monopodal support exercises. In addition, some exercises had different progressive levels.

The variables VAS, joint balance in flexion and extension, strength in extension and the Timed-up and Go test were evaluated before and after each DN session. The remaining variables were evaluated before the first DN procedure and after the third DN session. All variables were measured 3 months after the last session.

### 2.7. Statistical Analysis

The statistical analysis of the data was carried out using PASW Advanced Statistics (SPSS Inc., Chicago, IL, USA), version 24.0. Data were reported as mean (standard deviation) and confidence intervals (CI 95%). Firstly, the normal distribution of variables was verified using the Shapiro–Wilk test after a descriptive analysis. Levene’s test was used to assess the homogeneity of variances. Linearity was assessed by bivariate dispersion graphics of residual values observed from the expected values. Comparisons between groups were made for demographic and clinical data of reference using Student’s *t* test for continuous variables and Pearson’s chi-square test for categorical variables. All analyses followed the intention-to-treat principle, and the groups were analyzed as randomized.

Differences in measurements were detected by an analysis of variance of repeated measures (ANOVA) 2 × 3. A mixed analysis of variance (ANOVA) was employed to evaluate group × time interactions*, including the effect of time (baseline, three weeks after treatment and at six-month follow-up) as an intra-subject factor and group effects (MDR group versus EFT group) as inter-subject factors. Eta square (η^2^) was used to calculate the effect size (small when 0.01 ≤ η^2^ ≤ 0.06; medium when 0.06 ≤ η^2^ > 0.14; large when η^2^ > 0.14). Statistical significance was determined at *p* < 0.05.

## 3. Results

**Figure 2:**
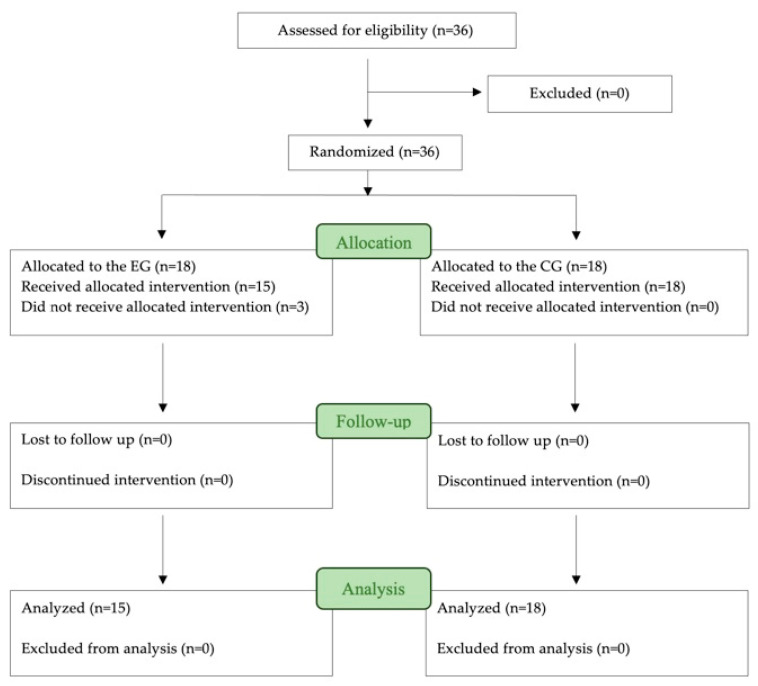
Flow chart of participants.

A total of 36 subjects, with ages between 24 and 65, were selected for the trial. After the inscription phase, the final sample included 33 participants (*n* = 33) (Figure 2): 8 men and 25 women with a mean age of 49 (SD 14.61) years. Three participants chose not to take part in the study. Out of the knees treated, 15 were right (45%), while the remaining 18 were left (55%).

Employment status data of the participants were collected: 3 of them were students, 6 worked in administration, 17 worked in other unspecified services, 6 were pensioners and 1 was unemployed. In the EG, there was 1 student, 1 administration worker, 12 other services workers, 4 pensioners and no unemployed individuals; in the CG, there were 2 students, 5 administration workers, 5 in other services, 2 pensioners and 1 unemployed individual. Regarding medication, 67% (10/15) of the EG subjects were taking some medication, as were 67% (12/18) of the CG subjects.

The baseline demographic characteristics (age, sex, height, weight and body mass index), clinical characteristics (level of functionality and self-perceived pain) and ROM (flexion and extension) are shown in Table 1. No inter-group statistically significant differences were found for any of the variables (*p* > 0.05 for all).

Table 2 presents the baseline, final and three-month follow-up measurements, as well as the between-group and intra-group mean differences. Statistically significant differences were observed, favoring the experimental group in terms of pain perception (VAS: F_1,31_ = 198.91 [*p* = 0.000] η^2^ = 0.86; DN4: F_1,31_ = 53.36 [*p* = 0.000] η^2^ = 0.63; WOMAC pain: F_1,31_ = 94.87 [*p* = 0.000] η^2^ = 0.75; WOMAC stiffness: F_1,31_ = 54.17 [*p* = 0.000] η^2^ = 0.63; WOMAC functionality: F_1,31_ = 211.59 [*p* = 0.000] η^2^ = 0.87), knee disability (KUJALA: F_1,31_ = 486.60 [*p* = 0.000] η^2^ = 0.94; LEFS: F_1,31_ = 813.11 [*p* = 0.000] η^2^= 0.96; TUG: F_1,31_ = 552.23 [*p* = 0.000] η^2^ = 0.94; 6MWT: F_1,31_ = 3204.35 [*p* = 0.000] η^2^ = 0.99; TSK: F_1,31_ = 637.27 [*p* = 0.000] η^2^ = 0.95; Pain Catastrophizing Scale: F_1,31_ = 187.01 [*p* = 0.000] η^2^ = 0.85), and range of movement (flexion: F_1,31_ = 0.27 [*p* = 0.60] η^2^ = 0.009; extension: F_1,31_ = 0.47 [*p* = 0.499] η^2^ = 0.016; extension strength: F_1,31_ = 1.71 [*p* = 0.20] η^2^ = 0.052). No statistically significant differences were found between before- and after-treatment measurements regarding the use of basic analgesic drugs. Finally, it must be considered that no side effects were observed.

**Figure 3:**
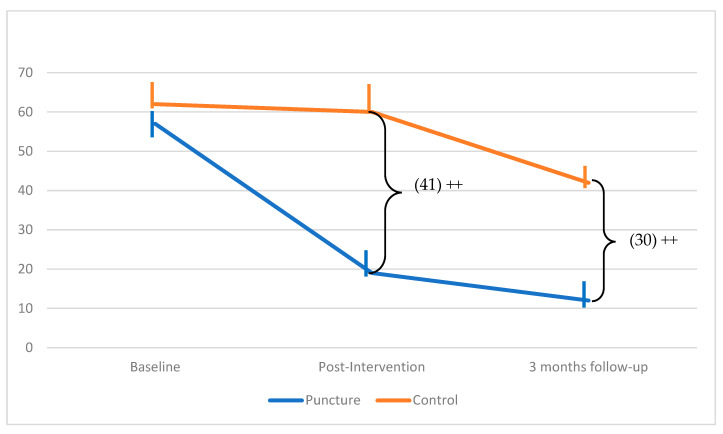
Between-group differences in pain intensity perceived along the treatments. ++: *p* < 0.001.

Figure 3 illustrates the between-group differences in the pain intensity perceived by the participants. No statistically significant differences were observed between the pre- and post-treatment measurements concerning the use of basic analgesic drugs. Finally, it is important to note that no side effects were observed.

## 4. Discussion

The main finding of this study was that DN of the popliteus muscle combined with an exercise program for patients with knee OA, compared to therapeutic exercise treatment alone, showed better results in pain, function and strength when compared to baseline data. It was also observed that the addition of DN improved pain catastrophizing and kinesiophobia at 3 months after the treatment. These are very relevant findings, since exercise has previously been shown to be very effective in improving the symptoms of the disease [42]. The results of this study show very interesting data regarding implementing DN of the popliteus muscle in addition to an exercise protocol for patients with knee OA.

Exercise has been shown to improve joint pain, function and quality of life in people with knee OA [10]. Despite the limited research available, exercise has been shown to be safe for articular cartilage in people with or at risk of this disease [43]. However, promoting adherence to exercise is sometimes difficult [44]; it is therefore important for professionals to supervise the implementation of these programs, suggesting strategies to increase adherence [42].

With this in mind, we hypothesized that DN of the popliteus muscle combined with therapeutic exercise for knee OA would provide better results in terms of function, strength and pain than therapeutic exercise alone. According to the results obtained in our study, pain decreased significantly (*p* < 0.001) when adding the DN technique to the exercise program, both in the VAS scale and in the WOMAC pain questionnaire, after the intervention. Sánchez-Romero’s study also showed statistically significant differences in NRS in the time factor (*p* < 0.0001), although there were no significant changes in the group–time interaction [28].

It is understood, according to the results of this study, that the combination of DN and therapeutic exercise obtained better results, in terms of pain reduction, than the performance of therapeutic exercises alone. The relationship with other studies on exercise for knee OA that also lasted months should be taken into account [28,45].

In terms of functionality, significant differences were observed between the experimental group and the control group, with improvements in the following variables: the WOMAC functionality scale, KUJULA score, LEFS questionnaire and 6MWT test. Thus, the addition of DN to the exercise program produces greater improvement in function than exercise alone. Mohammadreza Farazdaghi demonstrated significant improvements (*p* < 0.001) in functionality using DN in knee OA when compared to sham DN [16].

Extension strength also showed significant differences (*p* < 0.001) in this study, as well as the WOMAC stiffness scale after the intervention, unlike the results obtained in a 2018 pilot study in which no significant differences were observed between the time and group factor in this last variable, although it should be noted that the DN and exercise group did obtain significant improvements (*p* < 0.05) in the time factor, in 12 weeks of treatment, as opposed to the group that performed only therapeutic exercise [28].

Neuropathic pain measured with the DN4 questionnaire, the Pain Catastrophizing Scale and kinesiophobia, with the TSK-11 assessment, presented significant differences between groups in the observed data. It is believed that the improvement in functionality may be due to the relationship between pain and kinesiophobia [46], since the presence of kinesiophobia has been observed among patients with musculoskeletal pain [47].

Physical exercise is undoubtedly an important treatment in subjects with OA of the knee in both groups. Although better results were observed in the DN group in most variables at the 3-month follow-up, unlike other studies that did not observe significant improvements by adding this technique to the performance of an exercise program, the DN group had better results in most variables at the 3-month follow-up [25].

For all these reasons, therapeutic exercise may be recommended as the first line of treatment, since, on many occasions [25,45,48], it has been shown to improve the symptomatology of subjects with this condition. Moreover, the addition of DN could also be effective in terms of symptomatology treatment, as has been demonstrated by several authors, such as Mohammadreza Farazdaghi [16], or by M.S. Corbett in a meta-analysis on the effect of acupuncture on short-term knee pain [49]. However, further studies combining both procedures are needed to demonstrate the efficacy of this technique in combination with exercise in patients with knee OA.

One of the limitations of this study is the lack of long-term follow-up to confirm the continuity of the observed improvements. It is convenient to point out the need for a larger sample of participants in order to obtain more conclusive results in the variables studied. Patients’ mistrust of DN of the popliteus muscle should be taken into account, resulting in a limitation for the application of the technique. It should be noted that, in this study, no adverse effects were found in any of the participants.

Future research is needed to observe the long-term results of the combination of DN of the popliteus muscle with therapeutic exercise. In addition, it would be convenient to evaluate the degree of satisfaction with the treatment in order to predict its clinical relevance.

This randomized clinical trial demonstrates significant medium-term improvements in functionality, strength, catastrophizing, pain and kinesiophobia in patients with knee OA by combining DN of the popliteus muscle and therapeutic exercise. It highlights the benefits of the addition of DN in the treatment of this disease and offers interesting data for future research along the same lines.

## 5. Conclusions

The combination of DN of the popliteus muscle and therapeutic exercise were more effective than therapeutic exercise alone in terms of pain, function, strength and kinesiophobia for patients with knee OA. The main finding of this study is that the addition of DN of the popliteus muscle to therapeutic exercise produces significant improvements with respect to the baseline data in pain intensity and functionality when compared to therapeutic exercise alone, especially after the intervention.

## Figures and Tables

**Table 1:** Baseline characteristics of participants in the study groups.

	Total Sample (N = 33)	Puncture Group (*n* = 15)	Control Group (*n* = 18)	*p* Value *
Mean age (years)	48 (14.61)	45 (13.89)	51 (15.06)	0.262
Height (cm)	167 (10.70)	166 (11.15)	167 (10.62)	0.232
Weight (kg)	74.2 (14.12)	77.5 (9.45)	71.6 (16.89)	0.821
Body mass index	26.7 (4.86)	28.1 (4.25)	25.4 (5.11)	0.110
VAS (mm)	60 (20.8)	57 (20.3)	62 (21.5)	0.454
DN4 (0–10)	3.6 (1.43)	3.9 (1.55)	3.4 (1.34)	0.408
WOMAC pain	9.8 (5.55)	9.3 (5.06)	10.3 (6.04)	0.591
WOMAC stiffness	4.4 (2.35)	3.8 (2.04)	4.8 (2.55)	0.215
WOMAC functionality	29.9 (12.87)	28.4 (14.05)	31.2 (12.06)	0.531
KUJALA (%)	59 (11.0)	58 (8.3)	59 (13.1)	0.731
LEFS (%)	49 (11.8)	51 (11.5)	49 (12.3)	0.653
FLEXION (°)	117 (12.45)	116 (13.71)	117 (11.71)	0.940
EXTENSION (°)	2 (4.15)	3 (4.92)	1 (3.34)	0.274
Extension strength (mmHg)	51.8 (14.99)	53.9 (17.11)	50.1 (13.22)	0.473
TUG (″)	10.01 (1.59)	10.13 (1.07)	9.91 (1.95)	0.690
6MWT (m)	490.1 (89.0)	487.8 (66.4)	492.1 (106.2)	0.894
TSK	31 (6.89)	29 (4.01)	32 (8.65)	0.210
Pain Catastrophizing Scale	22 (8.29)	20 (9.09)	24 (7.17)	0.121

Data are reported as mean (SD); * between-group statistical significance (one-factor ANOVA).

**Table 2:** Mean score changes of knee pain and lower extremity function at baseline, post-intervention and follow-up.

	Groups	Baseline	Post-Intervention	Follow-Up (3 Months)	Pre-/Post-Differences	Between-Group Mean Changes Post-Intervention	Post-Intervention/Follow-Up Differences	Between-Group Mean Changes at Follow-Up
VAS (mm)	Puncture	57 ± 20.3	19 ± 8.83	12 ± 10.14	38 [27.4/47.2] **	41 [28.2/54.1] ^††^	7 [3.4/11.2] *	30 [19.8/40.5] ^††^
	Control	62 ± 21.5	60 ± 23.12	42 ± 17.33	2 [−3.2/6.6]		18 [6.3/30.3] *	
DN4 (0–10)	Puncture	3.9 ± 1.55	1.8 ± 1.37	0.3 ± 0.72	2.1 [1.2/2.9] **	0.4 [-0.6/1.4]	1.5 [0.8/2.1] **	1.8 [0.8/2.6] ^††^
	Control	3.4 ± 1.34	2.2 ± 1.59	2.1 ± 1.58	1.2 [0.7/1.6] **		0.1 [-0.4/0.7]	
WOMAC pain	Puncture	9.3 ± 5.06	3.7 ± 2.57	2.8 ± 1.69	5.5 [3.8/7.2] **	7.7 [4.3/10.9] ^††^	0.9 [0.01/1.9]	5.6 [3.4/7.8] ^††^
	Control	10.3 ± 6.04	11.4 ± 6.24	8.4 ± 3.83	−1.1 [−1.7/−0.3] *		3.0 [1.3/4.6] *	
WOMAC stiffness	Puncture	3.8 ± 2.04	0.5 ± 0.83	0.7 ± 0.90	3.3 [2.5/4.2] **	3.9 [2.4/5.4] ^††^	−0.2 [−0.5/0.2]	2.2 [1.2/3.3] ^††^
	Control	4.8 ± 2.55	4.4 ± 2.77	2.9 ± 1.76	0.4 [−0.2/0.9]		1.5 [0.5/2.4] *	
WOMAC functionality	Puncture	28.4 ± 14.05	14.2 ± 4.84	10.2 ± 4.76	14.2 [7.6/20.7] **	13.1 [7.4/18.8] ^††^	4.0 [0.1/7.9] *	14.3 [8.1/20.2] ^††^
	Control	31.2 ± 12.06	27.3 ± 9.85	24.5 ± 10.95	3.9 [1.0/6.8] *		2.8 [−0.9/6.6]	
KUJALA (%)	Puncture	58 ± 8.3	73 ± 11.77	77 ± 11.29	−15 [−19.3/−10.7] **	−16 [−28.1/−3.6] ^†^	−4 [−7.2/0.1]	−29 [−41.1/−15.7] ^††^
	Control	59 ± 13.10	57 ± 20.62	48 ± 21.75	2 [−5.1/9.5]		9 [2.0/15.8] *	
LEFS (%)	Puncture	51 ± 11.5	61 ± 11.46	68 ± 10.75	−10 [−14.5/−5.8] **	−18 [−27.5/−8.4] ^†^	−7 [−10.0/−4.5] **	−26 [−35.1/−18.5] ^††^
	Control	49 ± 12.3	43 ± 15.07	42 ± 12.54	6 [1.8/10.1] *		1 [−7.3/10.4]	
Flexion (º)	Puncture	116 ± 13.71	122 ± 9.95	123 ± 7.09	−6 [−8.8/−1.8] *	−1 [−7.8/6.2]	−1 [−3.6/2.5]	−2 [−7.2/3.0]
	Control	117 ± 11.71	121 ± 9.82	121 ± 7.36	−4 [−7.8/−0.4] *		0 [−2.8/4.2]	
Extension (º)	Puncture	3 ± 4.92	2 ± 3.16	1 ± 1.87	1 [−0.3/2.9]	0 [−2.4/2.3]	1 [0.1/2.9]	0 [−1.4/2.1]
	Control	1 ± 3.34	2 ± 3.48	1 ± 2.74	−1 [−1.3/0.2]		1 [−0.7/2.3]	
Extension strength (mmHg)	Puncture	53.9 ± 17.11	67.0 ± 17.25	73.5 ± 16.62	−13.1 [−19.6/−6.5] *	−15.4 [−26.6/−4.1] ^†^	−6.5 [−10.7/−2.3] *	−23.2 [−33.6/−12.8] ^††^
	Control	50.1 ± 13.22	51.6 ± 14.37	50.3 ± 12.65	−1.5 [−5.3/2.3]		1.3 [−5.7/8.2]	
TUG (″)	Puncture	10.13 ± 1.07	5.74 ± 1.85	5.66 ± 1.53	4.39 [3.3/5.4] **	4.06 [2.6/5.5] ^††^	0.08 [−0.4/0.5]	1.05 [−0.2/2.3]
	Control	9.91 ± 1.95	9.80 ± 2.12	6.71 ± 2.09	0.09 [−0.6/0.8]		3.09 [1.9/4.2] **	
6MWT (m)	Puncture	487.8 ± 66.4	558.4 ± 63.56	618.0 ± 35.75	−70.6 [−95.6/−45.5] **	−47.9 [−101.2/5.4]	−59.6 [−85.9/−33.2] **	−83.9 [−119.6/−48.1] ^††^
	Control	492.1 ± 106.2	510.5 ± 82.95	534.1 ± 59.31	−18.4 [−51.6/14.7]		−23.6 [−57.8/10.6]	
TSK	Puncture	29 ± 4.01	20 ± 4.93	18 ± 4.42	9 [5.9/13.4] **	10 [6.4/13.9] ^††^	2 [−0.4/3.3]	8 [3.8/12.6] ^††^
	Control	32 ± 8.65	30 ± 5.50	26 ± 7.22	2 [−0.1/5.0]		4 [1.2/5.6] *	
Pain Catastrophizing Scale	Puncture	20 ± 9.09	13 ± 8.61	4 ± 4.20	7 [4.6/9.4] **	12 [6.4/17.5] ^††^	9 [5.1/12.5] **	15 [10.9/19.8] ^††^
	Control	24 ± 7.17	25 ± 6.97	19 ± 7.54	−1 [−2.3/1.5]		6 [2.8/7.8] **	

Data are reported as mean ± SD or [95% confidence level]. * Indicates statistically significant intra-group differences (*p* < 0.05). ** Indicates statistically significant intra-group differences (*p* < 0.001). ^†^ Indicates statistically significant between-group differences (*p* < 0.05). ^††^ Indicates statistically significant between-group differences (*p* < 0.001).

## Data Availability

Data of this study are available upon reasonable request to the corresponding author.
